# The influence of refined enteral nutrition intervention on nutritional status and feeding intolerance in critically ill stroke patients

**DOI:** 10.3389/fneur.2026.1860690

**Published:** 2026-08-05

**Authors:** Neng Huang, Cailing Huo, Yutong Zhu, Jing He, Cheng Huang, Lan Zheng

**Affiliations:** 1 Rehabilitation Medicine Center and Institute of Rehabilitation Medicine, West China Hospital, Sichuan University, Chengdu, Sichuan, China; 2 West China School of Nursing, Sichuan University, Chengdu, Sichuan, China; 3 Key Laboratory of Rehabilitation Medicine in Sichuan Province, Chengdu, Sichuan, China

**Keywords:** critically ill patient, enteral nutrition, feeding intolerance, refined intervention, stroke

## Abstract

**Background:**

Critically ill stroke patients frequently experience dysphagia, posing challenges to adequate nutrient intake. Furthermore, these patients often remain in a prolonged state of high stress, hypermetabolism, and reduced immunity. While early enteral nutritional support is crucial, feeding intolerance remains a prevalent issue. This study evaluates the real-world impact of a refined enteral nutrition (EN) intervention on EN outcomes.

**Objective:**

To investigate the effect of a refined enteral nutrition intervention on nutritional status and feeding intolerance in critically ill stroke patients.

**Methods:**

This quasi-experimental study employed a pre-post design. The control group (*n* = 81; January–December 2022) was retrospectively enrolled, and the intervention group (*n* = 81; January–December 2023) was prospectively enrolled. The Control group received routine nutrition management, and the Intervention group received refined enteral nutrition management. Primary outcomes assessed included: (1) Nutritional markers: Serum albumin, hemoglobin, triceps skinfold thickness (non-paralyzed side), mid-upper arm circumference (non-paralyzed side); (2) EN intolerance incidence: Diarrhea (≥3 stools/day), vomiting, abdominal distension, elevated gastric residual volume (≥250 mL); (3) Disease severity: APACHE II score. Energy and protein targets were calculated individually (25–30 kcal/kg/day and 1.2–2.0 g/kg/day). Achievement of ≥70% of both targets was considered adequate. (4) Nutritional Risk Screening (NRS2002) and hospital length of stay were also recorded.

**Results:**

Post-intervention albumin levels were significantly higher in the Intervention group (40.69 ± 1.88 g/L) compared to the Control group (38.92 ± 1.21 g/L; *t* = −7.147, *p* < 0.001), mean difference 1.77 g/L [95% CI: 1.29–2.25], Cohen’s *d* = 1.12, with comparable baseline values (36.97 ± 2.34 vs. 36.81 ± 1.67 g/L; *p* = 0.056). Post-intervention hemoglobin levels were also significantly higher in the Intervention group (114.40 ± 8.00 g/L vs. 110.57 ± 6.52 g/L; *t* = −3.337, *p* = 0.001), despite comparable baseline values (97.37 ± 9.89 vs. 99.77 ± 5.92 g/L; *t* = 1.870, *p* = 0.630). Post-intervention triceps skinfold thickness was significantly greater in the Intervention group (12.29 ± 2.08 mm vs. 11.20 ± 2.40 mm; *t* = −3.087, *p* = 0.002), with comparable baselines (9.97 ± 2.10 mm vs. 9.82 ± 2.21 mm; *t* = −0.448, *p* = 0.655). Similarly, post-intervention mid-upper arm circumference was significantly higher in the Intervention group (21.11 ± 1.47 cm vs. 19.86 ± 1.65 cm; *t* = −5.068, *p* < 0.001), with comparable baseline values (17.15 ± 1.22 cm vs. 16.99 ± 1.37 cm; *t* = −0.762, *p* = 0.447). Post-intervention NRS2002 scores were significantly lower in the Intervention group (4.56 ± 0.82 vs. 5.07 ± 0.83; *p* < 0.001). Moreover, the APACHE II score was significantly lower in the Intervention group (15.06 ± 2.89) than in the Control group (19.85 ± 3.36; *t* = 9.236, *p* = 0.000). In the Intervention group, 91.4% of patients achieved ≥70% of energy targets and 88.9% achieved ≥70% of protein targets, compared to 64.2% and 59.3% in the Control group, respectively (both *p* < 0.001). The Intervention group also had a significantly shorter hospital stay (42.22 ± 7.25 vs. 44.78 ± 5.21 days; *p* = 0.011).

**Conclusion:**

The refined enteral nutrition intervention was associated with improved short-term nutritional status and reduced incidence of feeding intolerance in critically ill stroke patients. However, due to the quasi-experimental design historical controls, the inclusion of adjunctive therapies with unproven efficacy, and the lack of long-term outcome data (including 30-day mortality and functional recovery), categorical claims about improved prognosis cannot be made. The observed benefits may be associated with the comprehensive nursing protocol rather than the refined EN intervention alone and our findings should be interpreted as demonstrating an association rather than a causal relationship. Large-scale, multicenter randomized controlled trials are needed to confirm these findings.

## 1 Introduction

Critically ill stroke patients frequently present with dysphagia, leading to impaired oral intake. Additionally, these patients often experience prolonged periods of high stress, hypermetabolism, and reduced immunity. Early and appropriate nutritional support is crucial in managing this population (1). EN is the preferred method for critically ill stroke patients due to its physiological benefits (2). However, enteral feeding intolerance (EFI), primarily caused by gastrointestinal dysfunction (3, 4), is highly prevalent. Reported EFI incidence rates are 58.7% in international ICU populations and 73.6% in China (5, 6). EFI manifests as diarrhea, abdominal distension, nausea/vomiting, and other symptoms, frequently resulting in inadequate nutrient delivery, prolonged hospitalization, and increased morbidity and mortality (7). Consequently, optimizing EN delivery for ICU stroke patients is essential to mitigate EFI risk. This study evaluated the impact of a systematically refined enteral nutrition intervention on nutritional status and feeding tolerance in ICU stroke patients, aiming to inform clinical strategies for EFI prevention.

Although certain adjunctive therapies such as acupuncture, abdominal massage, and infrared irradiation have shown potential benefits for gastrointestinal function in some studies, their efficacy remains insufficiently established according to major international guidelines (e.g., ESPEN, SCCM/ASPEN). These therapies were included as part of routine institutional practice in the intervention group, not as independent study variables. Their potential influence on the observed outcomes is addressed in the limitations section.

## 2 Subjects and methods

### 2.1 Study design

This quasi-experimental study employed a pre-post design to evaluate the impact of refined enteral nutrition management. The control group (pre-intervention cohort) was retrospectively enrolled (January–December 2022), and the intervention group (post-intervention cohort) was prospectively enrolled (January–December 2023). Participants were not randomized; however, baseline comparability between groups was confirmed (Table 1). Temporal changes in practice may introduce bias, and this design limitation is addressed in the Discussion. Sample size was calculated based on the primary outcome (serum albumin). Assuming a clinically meaningful difference of 1.5 g/L (SD = 2.5) between groups, a two-sided alpha of 0.05, and a power of 90%, a minimum of 78 patients per group was required. To account for a potential 5% dropout rate, 81 patients were enrolled in each group. The study was reviewed and approved by the Biomedical Research Ethics Committee (2022–1846) of West China Hospital of Sichuan University, and it was conducted in accordance with the Declaration of Helsinki and its contemporary amendments, and the principles of good clinical practice. All participants signed an informed consent.

**Table 1:** Comparison of general information between two groups.

Items	Intervention group	Control group	Test statistic	*p* value
Age, mean ± SD	59.01 ± 12.81 (range 30–78)	57.90 ± 11.87 (range 23–83)	*t* = 0.573	0.286
Gender				
Male	42 (51.85%)	45 (55.56%)	*X*^2^ = 0.223	0.376
Female	39 (48.15%)	36 (44.44%)		
Course of disease	3.27 ± 0.82 (range 2–5)	3.23 ± 0.76 (range 1–6)	*t* = 0.322	0.748
Type of disease				
Ischemic	44 (54.32%)	43 (53.09%)	*X*^2^ = 0.025	0.500
Hemorrhagic	37 (45.68%)	38 (46.91%)		

### 2.2 Subjects

Critically ill stroke patients admitted between January 2022 and December 2023 were assigned to two cohorts: a retrospective Control group (*n* = 81; January 2022–December 2022) and a prospective Intervention group (*n* = 81; January 2023–December 2023). Setting and population characteristics: This study was conducted in a rehabilitation-oriented step-down unit of a tertiary hospital. All enrolled patients had survived the acute phase of stroke and were admitted for rehabilitation. None of the patients required mechanical ventilation during their stay, and the in-hospital mortality rate was 0%. Therefore, this population is distinct from traditional ICU cohorts, and outcomes such as mortality and ventilator-associated pneumonia are not applicable endpoints. Inclusion Criteria: (1) Diagnosis of stroke according to the *Chinese Guidelines for Diagnosis and Treatment of Acute Ischemic Stroke (2018)* (8), confirmed by brain computed tomography (CT) or magnetic resonance imaging (MRI). (2) Initiation of EN support therapy for the first time during the current admission. (3) Ability to cooperate with treatment protocols, with written informed consent provided by the patient or legally authorized representative. Exclusion Criteria: Pre-existing gastrointestinal dysfunction (e.g., diarrhea, nausea/vomiting, abdominal distension, or gastric retention) upon admission. (2) Significant hepatic impairment (e.g., transaminase elevation >3 times upper limit of normal) or severe concurrent systemic illness (e.g., acute cardiac failure, severe arrhythmia). (3) Contraindications to EN, including intestinal obstruction, intestinal fistula, intractable vomiting/diarrhea, recent gastrointestinal surgery (<4 weeks), or other conditions compromising EN safety. (4) Incomplete medical records precluding data analysis.

### 2.3 Methods

#### 2.3.1 Control group

Patients in the control group received standard nursing care. This included: (1) Optimal patient positioning for comfort and safety upon admission. (2) Continuous condition monitoring, including regular neurological assessments. (3) Vital sign measurement (e.g., blood pressure, pulse, respiratory rate) at 2-h intervals. (4) Maintenance of a clean, comfortable bed environment. (5) Comprehensive health education and rehabilitation guidance provided to patients and their families.

#### 2.3.2 Intervention group

Patients in the intervention group received a refined enteral nutrition protocol delivered through multidisciplinary collaboration (physicians, nurses, dietitians). This standardized approach included: timely EN initiation, systematic feeding tolerance monitoring, and individualized nutrition care planning.

##### 2.3.2.1 Refined enteral nutrition intervention structure and training

A multidisciplinary nutrition support team (physicians, nurses, dietitians) was established. Weekly standardized training sessions implemented evidence-based neurocritical care nutrition protocols (Table 2). EN formulations were individualized according to patients’ clinical and physiological parameters. EN support was initiated within 24 h of vital sign stabilization.

**Table 2:** Refined enteral nutrition member responsibilities.

Role	Responsibilities	Training content
Doctors	EN indication assessment	Management of swallowing difficulties in stroke, application of acupuncture and physiotherapy for enteral nutrition intolerance, etc.
Nurses	GRV monitoring and intolerance screening	Bedside ultrasound is used for gastric volume, position management, nasogastric tube care, etc.
Dietitians	Formula customization	Stroke-specific nutrient needs

##### 2.3.2.2 Refined enteral nutrition intervention protocol

The enteral nutrition protocol was individualized according to patients’ laboratory parameters, clinical status, and nutritional requirements. Feeding route (nasogastric/nasojejunal) and administration mode (continuous/intermittent) were determined accordingly. During administration: (1) Positioning: Head-of-bed elevation maintained at 30°–45°; (2) Infusion Method: Continuous 24-h delivery via infusion pump with controlled infusion rates; (3) Gastric Residual Volume (GRV) Monitoring: To proactively manage feeding tolerance, a graded approach was used. When the GRV was between 100 mL and 250 mL, the infusion rate was decreased as a preventive measure. However, if GRV reached or exceeded 250 mL, enteral nutrition was temporarily paused for 1 h, GRV rechecked, and prokinetic therapy administered. Infusion was resumed only if GRV decreased below 200 mL (9, 10); (4) Prokinetic Therapy: Prokinetic agents administered throughout the 14-day feeding period; (5) Feeding Position: Semi-Fowler’s position maintained during administration; (6) Tube Maintenance: Flushing with sterile warm water (37–40 °C) post-administration.

**Figure 1:**
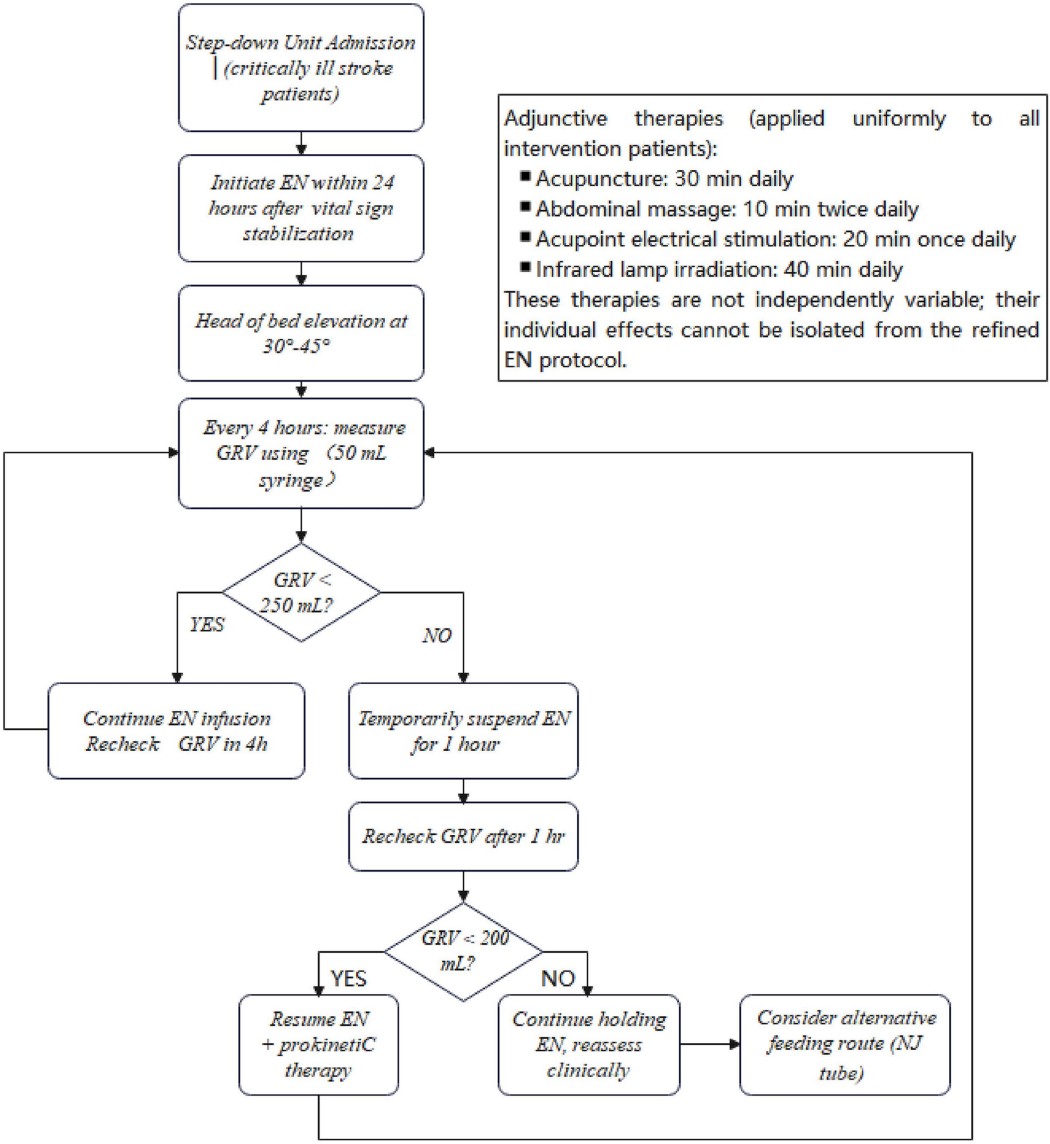
Decision-making algorithm for the refined enteral nutrition intervention protocol.

The decision-making algorithm for GRV monitoring and EN adjustment is illustrated in Figure 1.

##### 2.3.2.3 Medication administration

Dosing regimen was adjusted regularly based on patients’ biochemical and body conditions. Operators were familiar with the preservation of drugs used in enteral nutritional support. Appropriate nutritional and microecological preparations were applied. Drugs that improve gastric power, such as domperidone and mosapride, etc., were reasonably used.

##### 2.3.2.4 Individualized management

Specific challenges encountered in the management of individual patients were addressed through consultation with specialist staff, which facilitated development of individualized programs.

##### 2.3.2.5 Refined intervention of enteral feeding intolerance

EFI was assessed through 4-hourly gastric residual volume monitoring. To facilitate early EFI identification, medical staff received specialized training in intolerance recognition protocols. This training enabled timely detection of adverse reactions and accurate EFI assessment. Daily oral care was performed. Morning exercise regimens—including positional changes, active exercises, and passive mobilization—were implemented to reduce risks of gastroesophageal reflux, aspiration, and diarrhea, while expediting achievement of nutritional targets. Additionally, Traditional Chinese Medicine (TCM) therapies were administered uniformly to all 81 patients in the intervention group (no subgroup differentiation): (1) Daily 30-min acupuncture at specific acupoints (Zusanli, Zhongwan, Neiguan) (11); (2) Abdominal massage for 10 min twice daily (12); (3) Acupoint electrical stimulation for 20 min once daily (13); (4) Infrared lamp irradiation over the abdomen for 40 min daily to promote blood circulation and stimulate intestinal peristalsis. These interventions targeted gastrointestinal symptom improvement (bloating, vomiting, diarrhea) and EFI mitigation. It should be emphasized that these adjunctive TCM therapies are not standardized components of major international critical care nutrition guidelines (e.g., ESPEN, SCCM/ASPEN). They were implemented as part of routine institutional practice, and their independent contribution to the observed outcomes cannot be quantified. Because these therapies were applied to all intervention patients uniformly, no subgroup analysis can distinguish their effects from the refined EN protocol itself.

##### 2.3.2.6 Physiotherapy

The abdomens of patients were irradiated with Infrared baking lamp (40 min) to promote blood circulation, and vibrations were applied to stimulate intestinal peristalsis.

##### 2.3.2.7 Energy and protein targets

Energy and protein targets were calculated individually based on 25–30 kcal/kg/day and 1.2–2.0 g protein/kg/day, using ideal body weight. Actual delivery was recorded daily. Achievement of ≥70% of both energy and protein targets was considered adequate nutritional delivery.

### 2.4 Observation indicators

(1) Nutritional markers: Serum albumin (g/L), hemoglobin (g/L), triceps skinfold thickness (mm, non-paralyzed side), and mid-upper arm circumference (cm, non-paralyzed side) were measured on admission and before discharge; (2) Feeding intolerance: After enteral nutritional support, feeding intolerance was assessed, including diarrhea (≥3 bowel movements per day), nausea or vomiting, abdominal distension, and gastric retention (gastric residual volume ≥250 mL by syringe aspiration after 6 h of continuous infusion) (14); (3) Disease severity: The Acute Physiology and Chronic Health Evaluation II (APACHE II) score (range 0–71; score >15 indicates severe illness) was used to assess baseline disease severity and confirm comparability between groups. This score was not intended for longitudinal evaluation of intervention effects. This score comprises acute physiology score (APS), age score, and chronic health score, with higher scores associated with increased risk of hospital mortality (15). Of note, the Sequential Organ Failure Assessment (SOFA) score was not used, as its respiratory component (PaO_2_/FiO_2_ ratio) is not applicable to rehabilitation-oriented stroke patients who are not mechanically ventilated. (4) Nutritional risk screening: The Nutritional Risk Screening 2002 (NRS2002) score was calculated at admission and before discharge. NRS2002 includes three components: impaired nutritional status (0–3 points), disease severity (0–3 points), and age (≥70 years: 1 point) (16). A total score ≥3 indicates nutritional risk requiring nutritional support.

### 2.5 Statistics

Data were analyzed using SPSS 18.0. Normality of continuous variables was assessed using the Shapiro–Wilk test. Variables following a normal distribution (*p* > 0.05) were reported as mean ± standard deviation and compared using the independent-samples *t*-test (with Levene’s test for homogeneity of variances; Welch’s *t*-test used when variances were unequal). Effect sizes (Cohen’s *d*) and 95% confidence intervals (CIs) were calculated for key outcomes where applicable. Variables not following a normal distribution were reported as median (interquartile range, IQR) and compared using the Mann–Whitney U test. Categorical variables were expressed as frequencies and percentages and compared using the *χ*^2^ test or Fisher’s exact test, as appropriate. All statistical tests were two-sided, with a significance level of *α* = 0.05.

## 3 Results

### 3.1 Comparison of general data between two groups

The control group comprised 45 males and 36 females (mean age: 57.90 ± 11.87 years; range: 23–83) with a mean disease duration of 3.23 ± 0.76 years (range: 1–6). Stroke subtypes included ischemic stroke (*n* = 43) and hemorrhagic stroke (*n* = 38). Disease stages were distributed as follows: hyper-acute (*n* = 18), acute (*n* = 22), and recovery (*n* = 41). The intervention group included 42 males and 39 females (mean age: 59.01 ± 12.81 years; range: 30–78) with a mean disease duration of 3.27 ± 0.82 years (range: 2–5). Stroke subtypes consisted of ischemic stroke (*n* = 44) and hemorrhagic stroke (*n* = 37). Disease stage distribution was: hyper-acute (*n* = 14), acute (*n* = 22), and recovery (*n* = 45). No statistically significant between-group differences were observed in baseline characteristics (all *p* > 0.05), confirming comparability (Table 1).

### 3.2 Comparison of nutritional status between two groups

Both groups demonstrated significant improvements in all nutritional parameters post-intervention (all *p* < 0.05). The intervention group showed significantly greater improvement across all measures compared to controls (*p* < 0.05 for between-group differences). Baseline nutritional status did not differ significantly between groups (*p* > 0.05). Although serum albumin improved significantly in the Intervention group, this finding should be interpreted cautiously as albumin is influenced by inflammation and fluid status in addition to nutritional status. Post-intervention albumin difference: mean difference 1.77 g/L (95% CI: 1.29–2.25), Cohen’s *d* = 1.12, indicating a large effect. These findings indicate that the refined nutritional intervention more effectively enhances nutritional status and promotes anthropometric recovery (Table 3).

**Table 3:** Comparison of nutritional status between two groups.

Groups	*n*	Serum albumin (g/L)		Hemoglobin (g/L)		Triceps skinfold thickness (mm)		Triceps upper-arm circumference on the paralysis-free side (cm)	
		Pre-management	Post-management	Pre-management	Post-management	Pre-management	Post-management	Pre-management	Post-management
Intervention group	81	36.97 ± 2.34	40.69 ± 1.88^*^	97.37 ± 9.89	114.40 ± 8.00^*^	9.97 ± 2.10	12.29 ± 2.08^*^	17.15 ± 1.22	21.11 ± 1.47^*^
Control group	81	36.81 ± 1.67	38.92 ± 1.21^*^	99.77 ± 5.92	110.57 ± 6.52^*^	9.82 ± 2.21	11.20 ± 2.40^*^	16.99 ± 1.37	19.86 ± 1.65^*^
*t*		−0.483	−7.147	1.870	−3.337	−0.448	−3.087	−0.762	−5.068
*p*		0.056	0.000	0.630	0.001	0.655	0.002	0.447	0.000

^*^Comparison with cohort prior to nutrition management, *p* < 0.05.

### 3.3 Comparison of incidence of feeding intolerance between two groups

The intervention group demonstrated significantly lower incidence of diarrhea, nausea/vomiting, abdominal distension, and gastric retention compared to controls post-intervention (*p* < 0.05; Table 4).

**Table 4:** Comparison of incidence of feeding intolerance between two groups (%).

Groups	*n*	Diarrhea	Nausea/vomiting	Bloating	Stomach retention
Intervention group	81	8 (9.88%)	10 (12.35%)	3 (3.70%)	5 (6.17%)
Control group	81	25 (30.86%)	22 (27.16%)	10 (12.35%)	16 (19.75%)
*χ* ^2^		10.998	5.608	5.951	6.620
*p*		0.001	0.018	0.015	0.010

### 3.4 Comparison of management efficiency between two groups

APACHE II scores significantly decreased in both groups post-intervention (Intervention: 27.12 ± 2.99 to 15.06 ± 2.89; Control: 27.11 ± 2.71 to 19.85 ± 3.67; both *p* < 0.05). Baseline scores demonstrated group comparability (mean difference = 0.01 points). The intervention group achieved significantly lower final scores versus controls (15.06 ± 2.89 vs. 19.85 ± 3.67; between-group *p* < 0.05; mean difference 4.79 points [95% CI: 3.76–5.82], Cohen’s *d* = 1.45), representing a 4.8-point greater reduction compared to routine care. However, as APACHE II is not validated for longitudinal intervention effect assessment, these findings should be interpreted with caution and should not be construed as evidence of improved prognosis. This magnitude of improvement exceeds the established minimal clinically important difference for APACHE II, indicating substantial clinical impact (Table 5).

**Table 5:** Comparison of the management efficiency of the two groups of patients (%).

APACHE II	Intervention group (*n* = 81)	Control group (*n* = 81)	Test statistic	*p* value
Pre-management	27.12 ± 2.99	27.11 ± 2.71	*t* = −0.028	0.978
post-management	15.06 ± 2.89^*^	19.85 ± 3.67^*^	*t* = 9.236	0.000

^*^Comparison with cohort prior to nutrition management, *p* < 0.05.

### 3.5 Achievement of nutritional targets

In the Intervention group, 74/81 patients (91.4%) achieved ≥70% of energy targets and 72/81 (88.9%) achieved ≥70% of protein targets, compared to 52/81 (64.2%) and 48/81 (59.3%) in the Control group, respectively (both *p* < 0.001; Table 6).

**Table 6:** Comparison of nutritional target achievement between two groups.

Indicator	Intervention (*n* = 81)	Control (*n* = 81)	*χ* ^2^	*p*
Energy target ≥70%	74 (91.4%)	52 (64.2%)	17.34	0.000
Protein target ≥70%	72 (88.9%)	48 (59.3%)	18.92	0.000

### 3.6 Nutritional risk assessment

As shown in Table 7, baseline NRS2002 scores were comparable between groups (Intervention: 6.14 ± 0.73; Control: 6.19 ± 0.70; *p* = 0.657), indicating high nutritional risk in both cohorts. Post-intervention, both groups showed significant improvement (both *p* < 0.001 vs. baseline). However, the Intervention group achieved significantly lower NRS2002 scores compared to the Control group (4.56 ± 0.82 vs. 5.07 ± 0.83; mean difference −0.51, 95% CI:-0.77 to −0.25; *p* < 0.001; Cohen’s *d* = 0.62), indicating a greater reduction in nutritional risk.

**Table 7:** Comparison of NRS2002 scores between two groups.

Timepoint	Intervention (*n* = 81)	Control (*n* = 81)	*t*	*p*
Pre-management	6.14 ± 0.73	6.19 ± 0.70	0.445	0.657
Post-intervention	4.56 ± 0.82^*^	5.07 ± 0.83^*^	3.933	0.000

^*^Both groups showed significant improvement from baseline (both *p* < 0.001). Cohen’s *d* for post-intervention difference = 0.62.

### 3.7 Length of hospital stay

As this study was conducted in a rehabilitation-oriented step-down unit (secondary), all patients were clinically stable, none required mechanical ventilation, and in-hospital mortality was 0% in both groups. Ventilator-associated pneumonia did not occur in either group. The mean hospital length of stay was 42.22 ± 7.25 days in the Intervention group and 44.78 ± 5.21 days in the Control group. The Intervention group had a significantly shorter hospital stay, with a mean difference of 2.56 days (95% CI: 0.60 to 4.52 days; *t* = 2.581, *p* = 0.011; Cohen’s *d* = 0.41). ICU-specific length of stay was not separately recorded from total hospital stay, which reflects the rehabilitation-oriented nature of this unit where patients are admitted for comprehensive rehabilitation rather than acute critical care (Table 8).

**Table 8:** Comparison of hospital length of stay between two groups.

Item	Intervention (*n* = 81)	Control (*n* = 81)	*t*	*p*
Hospital length of stay (days)	42.22 ± 7.25	44.78 ± 5.21	2.58	0.011

In-hospital mortality was 0% and VAP did not occur in either group, as no patients required mechanical ventilation. ICU-specific length of stay was not separately recorded.

## 4 Discussion

### 4.1 Refined enteral nutrition intervention improves nutritional support in critically ill stroke patients

Critically ill neurostroke patients face heightened malnutrition risk due to disease complexity, impaired oral intake, and profound metabolic stress (17). The acute disease onset triggers a systemic stress response, frequently compromising gastrointestinal perfusion and leading to impaired digestion and absorption. Concurrently, reduced consciousness in most patients precludes voluntary feeding, resulting in prolonged gut inactivity and diminished food tolerance (18). While essential, conventional EN support often precipitates feeding intolerance (FI), further jeopardizing nutritional status (19). This study implemented a refined EN protocol specifically designed to optimize nutritional delivery and mitigate FI in stroke patients. Analysis demonstrated superior nutritional parameters in the intervention group versus controls, confirming that structured EN intervention significantly improves nutritional status in severe stroke. This evidence-based protocol, tailored to critical care realities, standardizes two key processes: EN initiation within 24 h, and systematic FI monitoring. Its scientific rigor effectively prevents FI and enhances EN efficacy (20). Crucially, this refined EN model provides a feasible, equipment-neutral implementation framework for stroke units, achievable through multidisciplinary coordination.

### 4.2 Refined enteral nutrition intervention effectively reduces feeding intolerance in critically ill stroke patients

EN serves as a vital nutritional support modality for stroke patients. While offering therapeutic benefits, EN is also associated with specific complications. Furthermore, limitations inherent in current medical models can constrain its therapeutic efficacy (21, 22). Notably, FI manifests earlier in stroke patients compared to those with other conditions (23). This study demonstrated a significantly lower incidence of FI in the Intervention group versus the Control group, indicating that the refined EN intervention effectively reduces FI. Addressing FI represents a critical priority within the refined EN protocol. The superior outcomes observed in the intervention group—significantly reduced FI incidence (Table 4) and a 4.6% improvement in albumin levels (*p* < 0.001)—confirm enhanced nutrient absorption. These findings align with established evidence that protocol-driven EN optimizes gastrointestinal tolerance (24). Within this study, patients exhibiting diverse FI symptoms received the refined intervention protocol, incorporating targeted measures such as prokinetic agents and physical interventions to promote gastric motility. For patients experiencing EN intolerance, collaborative communication among healthcare professionals is essential. This facilitates the development of individualized feeding regimens, ensuring EN support efficacy and preventing FI occurrence.

### 4.3 Refined enteral nutrition intervention may be associated with better short-term outcomes

Although APACHE II scores showed a statistically significant between-group difference post-intervention (15.06 ± 2.89 vs. 19.85 ± 3.67, *p* < 0.001), it is critical to note that APACHE II is not designed for dynamic monitoring of intervention effects (25, 26). First, APACHE II was originally developed and validated as a prognostic scoring system for predicting in-hospital mortality at the time of ICU admission, not as a tool for measuring changes in disease severity over time in response to therapeutic interventions (25, 26). The score’s components—acute physiology, age, and chronic health status—are intended to capture baseline risk, not treatment response. Second, APACHE II scores measured at different time points are not directly comparable for longitudinal assessment, as the score weights physiologic derangements that may fluctuate independently of the intervention being studied. Factors such as natural disease recovery, concurrent treatments, and resolution of acute phase response can all influence serial APACHE II measurements, making it impossible to attribute changes solely to the refined EN protocol. Third, the 4.79-point mean difference (95% CI: 3.76–5.82), while statistically significant, should be interpreted with caution. Although this difference exceeds the reported minimal clinically important difference (MCID) for APACHE II in some studies, the MCID was established for mortality prediction at admission, not for detecting clinically meaningful improvement from an intervention. Therefore, this difference reflects between-group baseline severity comparability rather than a causal effect of the intervention on prognosis. Fourth, in the absence of validated, stroke-specific severity scores (e.g., NIHSS) or long-term functional outcome measures (e.g., modified Rankin Scale at 30 or 90 days), APACHE II alone cannot serve as evidence of prognostic improvement. Our study did not collect these essential long-term outcome data, which represents a significant gap in our ability to make claims about prognosis. Therefore, the observed APACHE II difference should be interpreted solely as indicating that both groups were comparable at baseline and experienced clinical improvement over time, but it should not be construed as evidence that the refined EN intervention improved prognosis. The association between the intervention and APACHE II reduction warrants further investigation in properly designed randomized controlled trials with appropriate long-term outcome measures.

Therefore, this difference should not be interpreted as evidence of prognostic improvement. The 4.79-point mean difference (95% CI: 3.76–5.82), while statistically significant, reflects baseline severity comparability rather than a causal effect of the intervention on prognosis. In addition, the refined enteral nutrition intervention was associated with a significantly shorter hospital stay (mean difference 2.56 days; 95% CI: 0.60–4.52; *p* = 0.011; Cohen’s *d* = 0.41). While the absolute reduction of 2.56 days may appear modest, it is clinically meaningful in a rehabilitation setting where the average length of stay is approximately 44–55 days. This finding suggests that improved enteral nutrition management may contribute to faster recovery and earlier discharge, potentially reducing healthcare costs and bed occupancy. However, given the quasi-experimental design and the potential for confounding, this association should not be interpreted as causal. Implementation of refined enteral nutrition not only improves gastrointestinal function and enhances nutrient solution absorption but also effectively prevents the occurrence of feeding intolerance. This strategy improves patients’ nutritional status, avoids negative nitrogen balance, and may benefit short-term recovery (27).

Furthermore, the refined EN intervention was associated with a significantly greater reduction in NRS2002 scores compared to routine care (post-intervention difference: −0.51, 95% CI: −0.77 to −0.25, *p* < 0.001, Cohen’s *d* = 0.62). The NRS2002 is a validated nutritional risk screening tool that incorporates nutritional status, disease severity, and age. Both groups started with high nutritional risk (mean scores >6, where ≥3 indicates risk). While both groups improved significantly, the intervention group achieved a mean score of 4.56, approaching the threshold for normal nutritional status, whereas the control group remained at 5.07. This suggests that the refined EN protocol more effectively reduced nutritional risk, which may have contributed to the shorter hospital stay observed in the intervention group.

### 4.4 Limitations

This study has several limitations. First, the quasi-experimental pre-post design may introduce temporal bias, as changes in practice over time (e.g., staff experience, unit protocols) could have influenced outcomes. Second, the adjunctive therapies described (acupuncture, massage, electrical stimulation, infrared irradiation) are not internationally standardized according to major critical care nutrition guidelines (e.g., ESPEN, SCCM/ASPEN). These may have acted as unmeasured confounders, and their independent effects cannot be separated from the refined enteral nutrition protocol. Third, serum albumin was used as a nutritional marker despite being a negative acute-phase reactant. Changes in albumin may reflect resolution of inflammation rather than true nutritional improvement. We did not systematically adjust for inflammatory markers (e.g., CRP), liver function, renal function, or fluid status, which may have confounded the observed differences. Therefore, we have primarily interpreted improvements in hemoglobin and anthropometric measures as indicators of nutritional benefit. Fourth, the GRV threshold of ≥250 mL was applied as an absolute value without adjustment for infusion rate, which may not align with all published protocols. Finally, the study was conducted at a single tertiary hospital in China, which may limit generalizability to other healthcare settings with different resources, staffing models, or nutritional protocols. Sixth, ICU and hospital length of stay were not reliably captured for the retrospective cohort, precluding robust between-group comparisons. However, in-hospital mortality was 0% in both groups, indicating that the study population was relatively stable and admitted after the acute phase. Seventh, the SOFA score was not used because it is not appropriate for the rehabilitation-oriented stroke population; SOFA requires assessment of the PaO_2_/FiO_2_ ratio, which is not applicable to patients who are not mechanically ventilated. Therefore, APACHE II was used solely for baseline comparability, not as a longitudinal outcome measure. Its post-intervention difference should not be interpreted as prognostic improvement, as the score was originally developed for mortality risk prediction at admission, not for dynamic monitoring of treatment response (25, 26). Eighth, NRS2002 was used for nutritional risk screening rather than NUTRIC; while NRS2002 is validated in stroke settings, this limits direct comparison with studies using NUTRIC. Ninth, 30-day mortality and long-term functional outcomes (modified Rankin Scale, NIHSS) were not collected. Tenth, population specificity: This study was conducted in a rehabilitation-oriented step-down unit, where patients had survived the acute phase of stroke, were clinically stable, and did not require mechanical ventilation. The 0% in-hospital mortality rate and absence of VAP reflect this unique population. Therefore, the findings may not be generalizable to acute ICU settings, where patients are more critically ill and have higher baseline mortality risks. Comparisons with studies conducted in acute ICUs should be made with caution.

### 4.5 External validity

The study was conducted in a single tertiary hospital in China, which may limit generalizability to other healthcare systems with different resources, staffing, or nutritional protocols. However, the core components of the refined EN protocol (early initiation, systematic GRV monitoring, individualized energy and protein targets, and multidisciplinary collaboration) are adaptable to most intensive care settings. The adjunctive TCM therapies may not be available or accepted internationally, which further limits external validity. Future multicenter studies across diverse healthcare systems are needed to confirm the generalizability of these findings.

## 5 Summary

The refined enteral nutrition intervention was associated with improved short-term nutritional status and reduced the incidence of feeding intolerance in critically ill stroke patients. However, due to the quasi-experimental design with historical controls, the inclusion of adjunctive therapies with unproven efficacy, and the lack of long-term outcome data (including 30-day mortality and functional recovery), categorical claims about improved prognosis cannot be made. The observed benefits may be associated with the comprehensive nursing protocol rather than the refined EN intervention alone, and our findings should be interpreted as demonstrating an association rather than a causal relationship. Large-scale, multicenter randomized controlled trials are needed to confirm these findings and to evaluate the independent effects of specific intervention components.

## Data Availability

The original contributions presented in the study are included in the article/supplementary material, further inquiries can be directed to the corresponding author.
